# Sources of Pre‐ and Postnatal Maternal Energy Allocation to Offspring in a Long‐Lived, Capital Breeder: The Grey Seal (*Halichoerus grypus*)

**DOI:** 10.1002/ece3.73224

**Published:** 2026-03-16

**Authors:** M. Sanchez, W. D. Bowen, C. E. den Heyer, S. J. Iverson

**Affiliations:** ^1^ Department of Biology, Life Science Centre Dalhousie University Halifax Nova Scotia Canada; ^2^ Ocean Ecosystem Science Division Bedford Institute of Oceanography Dartmouth Nova Scotia Canada

**Keywords:** age, maternal energy allocation, offspring size, parity, pinniped

## Abstract

Parental energy allocation impacts the fitness of offspring and parents alike and should provide insight into the sources of variation in life history traits. Nevertheless, few studies have simultaneously assessed multiple sources of variation in maternal energy allocation and how allocation might vary over a female's lifetime. We used 20 years of cross‐sectional (females sampled once) and longitudinal (same female sampled more than once) data on 222 known‐age grey seals ( *Halichoerus grypus* ), a long‐lived capital breeder and 324 of their offspring to examine the influence on maternal energy allocation during lactation of the following variables: maternal postpartum mass (MPPM), total maternal mass loss (TMML), relative maternal mass loss (RMML = TMML:MPPM), mass transfer efficiency, lactation duration and pup birth and weaning mass. Mixed‐effect general linear models were used to examine the influence of predictors on response variables, with maternal identity and study year included as random effects. Our findings indicate that during the 17‐day lactation period, females expended about 37% (73 kg) of their MPPM to support their own metabolism requirements and milk production. Maternal age and MPPM had significant effects on multiple aspects of maternal energy allocation, including TMML, lactation duration, pup birth mass and pup weaning mass. Heavier females lost more body mass over lactation than lighter females. Maternal allocation increased throughout early life, plateaued in slow‐growing prime‐age females, and then declined in older females. Parity also affected maternal allocation, but the effect was limited to young females, with influences on pup birth mass, proportional pup mass gain and pup weaning mass. Females that lactated longer had greater RMML and produced pups with greater weaning mass. The effect of pup sex differed depending on MPPM, with lighter females experiencing greater TMML when nursing female pups than male pups and heavier females experiencing the reverse. Despite the substantial loss of body mass during lactation, 33 females measured in two consecutive years generally recovered fully from this energy expenditure, with no evidence of reduced allocation in the next year compared with the previous year. Prenatal allocation (pup birth mass) was a quadratic function of maternal age, increasing parity and pup sex, with female pups born lighter than males. Postnatal allocation (offspring weaning mass) also varied with maternal age, parity and pup sex, but TMML, lactation duration and pup birth mass were also influential.

## Graphical abstract

Few studies have simultaneously assessed multiple sources of variation in maternal energy allocation and how allocation might vary over a female's lifetime. We used 20 years of cross‐sectional and longitudinal data on 222 known‐age grey seals, which was collected as part of long‐term monitoring programme of grey seals that breed on Sable Island.

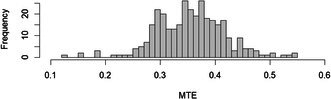

## 1 Introduction

Parental energy allocation impacts the fitness of offspring and parents alike (Smith and Fretwell 1974; Stearns 1992; Wolf and Wade 2001). Mothers that do not allocate sufficient energy to their offspring risk reducing their own fitness through lower offspring survival (Marshall and Keough 2008; Ozgul et al. 2010). Energy allocation to offspring can have long‐lasting consequences on offspring life histories and thus to their fitness as adults (Lindstrom 1999; Forchhammer et al. 2001; Rasanen and Kruuk 2007; Badger et al. 2023). As population dynamics of long‐lived species are often strongly influenced by variation in offspring survival (Gaillard et al. 2000; Ozgul et al. 2010), it is important to understand ecological and biological factors, such as maternal allocation, that affect this fitness component. Nevertheless, few studies have simultaneously assessed multiple sources of variation in maternal energy allocation and how allocation might vary over an individual's lifetime (Macdonald et al. 2020).

Maternal energy allocation to offspring should depend on the amount of food energy available to the mother (van Noordwijk and de Jong 1986; King et al. 2011), along with other traits such as breeding experience and age (Hamel et al. 2012). However, the relationship between maternal traits and maternal allocation often depends on the reproductive tactic used by the species. Mammals have developed two principal strategies along a continuum of resource use to support the high energetic costs of lactation (Drent and Daan 1980; Jonsson 1997). At one end of the continuum is income breeding, whereby females rely on food intake during lactation. At the other end is capital breeding, in which females fast throughout lactation and therefore must rely on stored body reserves obtained prior to giving birth to fuel their own metabolic requirements and support milk production. The costs and benefits of these resource‐use strategies differ depending on the timing and variability of resource availability (Boyd 2000; Stephens et al. 2014). But in capital breeders, fasting during lactation means that maternal resources are both finite and measurable (Smout et al. 2020), providing an ideal opportunity to measure the consequences of energy allocation for both mother and offspring.

In capital breeding species, maternal loss of body mass over lactation integrates both rate and duration of nutrient delivery. Differences in energy acquisition prior to the breeding season can result in variation in maternal postpartum mass (MPPM) and lipid stores at parturition that may impose constraints on energy allocation during lactation (McMahon et al. 2017). Such constraints influence both the magnitude and duration of maternal expenditure, which consists largely of total milk energy output (Mellish et al. 2000), which is in turn reflected by offspring body mass and growth (Crocker et al. 2001; Ronget et al. 2018). Pup growth and size at weaning have consequences for survival (McMahon et al. 2000; Hall et al. 2001; Bowen et al. 2015), and ultimately, the lifetime reproductive success of mothers (Trillmich 1996).

Although total maternal mass loss (TMML) is a useful measure of allocation, decomposing mass loss into the components of rate and duration of loss can provide additional insight as there may be a trade‐off between these two components. Mothers might adjust their allocation based on the environmental conditions they experience as the availability of food may vary. We expect this to be particularly true in capital breeding species, where females finance the delivery of nutrients to offspring entirely from body stores. Females that differ in body size may also differ in the amount of energy available for allocation (Mellish et al. 1999; Hamel et al. 2012). Thus, allocation strategies of capital breeders may be more dependent on maternal body size than income breeders (Festa‐Bianchet and Jorgenson 1998; Dobson et al. 1999; Boyd 2000).

There is ample evidence that maternal body mass is an influential trait with respect to energy allocation. However, relative allocation (i.e., accounting for differences in maternal size), should better measure allocation strategies in capital breeders than absolute maternal allocation (Hamel et al. 2012). Similarly, although offspring mass at weaning is often used as a measure of maternal allocation (Broussard et al. 2005; Bardsen et al. 2008; Hamel et al. 2011), the ratio of offspring mass to maternal mass should be more relevant to the fitness consequences of maternal allocation because of the strong relationship between the two and offspring survival (Ronget et al. 2018). However, this ratio is not commonly used as a measure of maternal allocation because of the difficulty of gathering sufficient mass data on mother–offspring pairs.

Grey seals (*Halicheorus grypus*) are long‐lived, iteroparous, capital breeders (Pomeroy et al. 1999; Bowen et al. 2006; Badger et al. 2021). Grey seals give birth to a single offspring, simplifying the investigation of allocation by eliminating variation in litter mass and number and the effects of differing offspring sex ratio within and among litters. Primiparity occurs as early as age 4 years and females continue to reproduce for several decades. They grow through their mid teens (Bowen et al. 2006), thus providing an opportunity for trade‐offs between maternal somatic growth and offspring allocation (Reznick 1985). Adult females store body energy mainly in the form of subcutaneous fat (i.e., blubber) in the months prior to arriving at the breeding colony (Beck et al. 2003), where they fast during the entire 16–20 days of lactation (Iverson et al. 1993). Previous research has shown that heavier females produce larger offspring (Iverson et al. 1993; Pomeroy et al. 1999; Bowen et al. 2006), but the combined effects of maternal and offspring traits on energy allocation have not been simultaneously examined.

Here we use 20 years of cross‐sectional and longitudinal data on known‐age females and their offspring to test hypotheses about the factors influencing components of maternal allocation. We examined allocation by first investigating the influence of maternal traits and then the associated effects of this allocation on offspring. To do this, we examined the factors that influence total allocation, expressed as TMML over the course of lactation and relative allocation (relative maternal mass loss, RMML), expressed at the ratio of TMML:MPPM. We also examined the factors influencing the duration of maternal allocation (i.e., lactation length).

Patterns of reproductive allocation within individuals may differ in the prenatal and postnatal periods (Lock et al. 2007; Weladji et al. 2010; Paterson et al. 2016) because these two periods reflect the different physiological processes of gestation and lactation (Oftedal 1985). This may be particularly true in capital breeding species since resource allocation is decoupled from food acquisition during the postnatal period. Our data on birth mass allowed us to investigate prenatal allocation of maternal resources, whereas our data on offspring weaning mass provide an opportunity to examine the consequence of post‐parturient allocation. We also calculated the ratio of mass gain by offspring relative to that lost by mothers to examine how the efficiency of nutrient transfer might vary with maternal and offspring traits. Finally, as lactation is the most energetically costly component of female reproduction (Gittleman and Thompson 1988), it should have a fitness cost if maternal body mass cannot be recovered before the next reproduction (Smout et al. 2020). Thus, we might expect in species where adult females have a high survival rate and multiple reproductive opportunities, such as grey seals (den Heyer and Bowen 2017), that the costs of reproduction will be expressed mostly as reduced maternal care (Festa‐Bianchet et al. 2019). We used data on females lactating in consecutive years to test whether the magnitude of previous allocation was associated with reduced allocation in the following year, as has been reported in other mammals (Creighton et al. 2009; Hamel et al. 2011; Hanson et al. 2019).

## 2 Materials and Methods

Our study was conducted on Sable Island (43.9337° N, 59.9149° W), Nova Scotia, Canada during the December–February breeding seasons from 1992 to 2011. Located along the outer edge of the Scotian Shelf, Sable Island is a crescent shaped, partially vegetated sand bar approximately 42.5 km in length. The number of pups born on the island each year increased over the course of our study from about 15,000 in 1992 to some 60,000 in 2011 (den Heyer et al. 2020), although local density of lactating females did not change as the available fraction of the island used by females also increased during the period of population growth.

We analysed 324 observations of body mass changes in 222 females and their offspring over the course of lactation. Thirty‐three of those females were sampled in consecutive years, permitting us to test whether previous energy allocation influenced energy allocation to offspring during the next breeding season. Study females had all been permanently and individually marked as pups shortly after they were weaned with a unique 3‐ or 4‐digit hot‐iron brand and were therefore of known age.

Pairs were captured using purpose‐built pole nets but not sedated. Females move only meters from the birth sites during the lactation (Allen et al. 2022) and allo‐suckling is rare in the Sable Island colony (McCulloch and Boness 2000) ensuring that we captured the female's biological offspring. Mothers were weighed using a 300‐kg capacity Dalton spring scale suspended from a tripod, whereas pups were weighed using a 100‐kg capacity scale suspended from a pole held by two researchers. Disturbance of study pairs lasted about 10–15 min for each capture. Although births were rarely observed, date of parturition was reliably determined to within 24 h during daily surveys by the presence of birth fluids and blood on the sand and on the female's hind flippers, presence of the placenta and the yellowish pelage of newly born pups. Mothers and their pups were weighed twice during lactation, at 3 days (sometimes ranging 4–5 days) and again at 10–12 days postpartum; pups were also weighed again at weaning. Pups of these known‐age females were marked with a semi‐permanent, uniquely numbered tag in the hind flipper to ensure the correct pup was weighed after mothers departed the colony at weaning. We chose to measure mass at least 3 days postpartum rather than at birth to allow for the uninterrupted development of the mother–pup bond, thereby greatly eliminating the risk of abandonment. Pup sex, maternal age, lactation duration (difference between weaning date and birth date) and parity were recorded. After the second capture, study females and their pups were visited daily, but not disturbed, to obtain an accurate date of weaning, defined as the day a female left the pup and departed the colony. Weaning mass was measured when the pup was first observed alone, usually on the day following the female's departure, and pup sex was confirmed at that time.

Based on previous work, we predicted MPPM, age and parity would influence maternal energy allocation (Mellish et al. 1999; Pomeroy et al. 1999; Bowen et al. 2006; Macdonald et al. 2020). Each female's MPPM was calculated by extrapolating her body mass at first capture to her parturition date using her measured rate of mass loss between the first and second capture. TMML during the lactation period was calculated as the difference between her estimated MPPM on the day of birth and her estimated mass at weaning, calculated using a female's individual rate of mass loss. This approach was warranted as Mellish et al. (1999) showed that maternal mass loss during lactation in our study population was constant over the lactation period. We also calculated RMML as the ratio of TMML to MPPM.

Parity can influence maternal allocation (Macdonald et al. 2020), with more experienced females allocating more to offspring regardless of MPPM. The first time a female was sighted in the breeding colony with a pup was considered her age at first birth. The estimated probability of sighting a female if she was alive on the island ranged from 0.8 to 0.95 between 1992 and 2016 (den Heyer and Bowen 2017). As sighting probability is less than 1.0 and grey seals do occasionally breed elsewhere (Bowen et al. 2015), it is possible that we could have missed the first birth of some females and therefore overestimated their age at first birth and underestimated parity. However, as 80% of females in the Northwest Atlantic population give birth on Sable Island (den Heyer et al. 2020), the impact of females breeding elsewhere on our estimate of parity is expected to be small. Parity was coded as 1 for primiparous or as 2 and 3+ for multiparous females. Pup birthdate also can influence maternal allocation through its effect on breeding colony density and harassment by other adults (Boness et al. 1995). However, we did not include birth date as a fixed effect in this study, as there was not sufficient variation in the birth dates of the females sampled.

Pup mass gain was calculated as the difference between the first measurement and weaning. We did not extrapolate the first measurement of pup mass to birth mass, as Mellish et al. (1999) found that pups gain mass at a lower rate in the first few days of lactation than later. For convenience, we refer to pup mass at first measurement as ‘birth mass’. Pup sex was included as a categorical covariate to account for the fact that male pups are heavier than female pups on average (Bowen et al. 2006). We also calculated mass transfer efficiency (MTE) as mass gained by the pup divided by the mass loss of its mother.

### 2.1 Statistical Models

**FIGURE 1:**
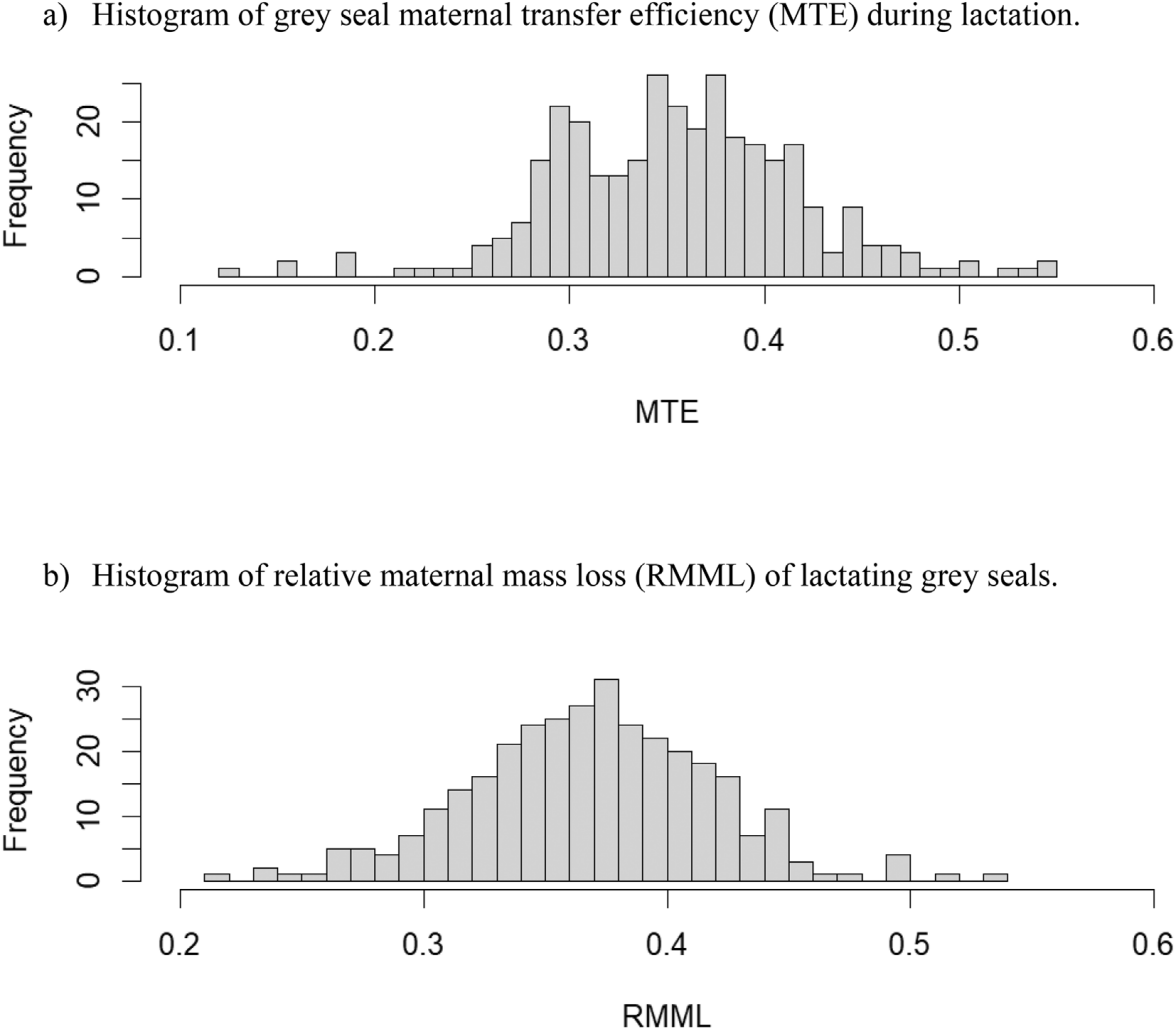
(a) Histogram of grey seal maternal transfer efficiency (MTE) during lactation. (b) Histogram of relative maternal mass loss (RMML) of lactating grey seals.

All statistical analyses were conducted in R version 4.4.1 statistical software (http://www.cran.r‐project.org/). Of the study females, 61 were measured in two to seven breeding seasons and 33 of these were measured in consecutive years. Mixed‐effect general linear models (GLMs) were used to examine the relationships between the response variables and predictors. Models were fit using the *lme4* (Bates et al. 2015) package in R version 1.3.959 (R Core Team 2020) and model selection was based on Akaike information criterion (AIC_c_), with smallest Δ AIC_c_ and highest AIC_c_ weights (w) (Burnham and Anderson 2002) taken as evidence for the preferred model. Seven response variables were used to examine patterns of maternal energy allocation and their associated effects on pup traits (Table 1). In addition to main effects, several interactions between covariates were examined: the interaction between maternal age and MPPM, between maternal age and parity, and between pup sex and MPPM. The only interaction retained in the final analysis was pup sex and MPPM in the TMML model. Each response variable was modelled with all possible combinations of the covariates (Table 1) using the package *MuMIn* (Bartoń 2020). While concerns have been raised about data exploration through all possible model combinations, (Burnham and Anderson 2002), in this case it is reasonable as we know from previous work that all selected variables could influence maternal allocation and therefore represent biologically plausible hypotheses. Goodness of fit of each model was assessed by examining the residuals from the *stats* package in R (R Core Team 2020). Continuous variables were centred and scaled to a mean of zero and standard deviation of 1 for model selection. Coefficients of the covariates in the best supported models were estimated by re‐running the analysis using the untransformed data and the magnitude of the fixed effects were illustrated using the *effects* package (Fox 2003). Conditional and marginal *R* ^2^ (Nakagawa and Schielzeth 2013), another measure of model fit, were calculated for each model using the *MuMIn* package (Bartoń 2020). Response variables for the proportional models were normally distributed (Figure 1) and therefore were modelled as linear mixed‐effects models rather than logistic models.

**TABLE 1:** Maximal models to test for the influence of maternal and pup traits on allocation response variables. Maternal identity and study year were included as random effects in all models.

Response variable	Fixed effects
MPPM (kg)	*β*_Female age_* Female age_ij_ + *β*_*Female age*_^*2*^ I(Female age_ij_^2^) + *β*_Pup sex_* Pup sex_ij_ + *β*_Parity_*Parity_ij_
TMML (kg)	*β*_Female age_* Female age_ij_ + *β*_*Female age*_^*2*^ I(Female age_ij_^2^) + *β*_MPPM_* MPPM_*ij*_ + *β*_Parity_*Parity_*ij*_ + *β*_Pup sex_* Pup sex_*ij*_ + *β*_MPPM*Pup sex_* MPPM* Pup sex
RMML (TMML/MPPM)	*β*_Female age_* Female age_ij_ + *β*_*Female age*_^*2*^ I(Female age_ij_^2^) + *β*_Pup sex_* Pup sex_ij_ + *β*_Pup birth mass_* Pup birth mass_ij_ + *β*_Pup age_* Pup age_ij_
MTE (pup gain/maternal loss)	*β*_Female age_* Female age_ij_ + *β*_*Female age*_^*2*^ I(Female age_ij_^2^) + *β*_Pup sex_* Pup sex_ij_ + *β*_Parity_* Parity_ij_ + *β*_MPPM_* MPPM_ij_
Lactation duration (d)	*β*_Female age_* Female age_ij_ + *β*_*Female age*_^*2*^ I(Female age_ij_^2^) + *β*_Pup sex_* Pup sex_ij_ + *β*_Parity_* Parity_ij_ + *β*_Pup birth mass_* Pup birth mass_ij_ + *β*_MPPM_* MPPM_ij_ + *β*_Pup age_* Pup age_ij_ + *β*_Pup growth rate_* Pup growth rate_ij_
Pup birth mass (kg)	*β*_Female age_* Female age_ij_ + *β*_*Female age*_^*2*^ I(Female age_ij_^2^) + *β*_Pup sex_* Pup sex_ij_ + *β*_MPPM_* MPPM_ij_ + *β*_Parity_* Parity_ij_ + *β*_Pup age_* Pup age_ij_
Pup weaning mass (kg)	*β*_Female age_* Female age_ij_ + *β*_*Female age*_^*2*^ I(Female age_ij_^2^) + *β*_Pup sex_* Pup sex_ij_ + *β*_Lactation duration_* Lactation duration_ij_ + *β*_Parity_*Parity_ij_ + *β*_Pup birth mass_* Pup birth mass_ij_ + *β*_TMML_* TMML_ij_

Abbreviations: MPPM, maternal postpartum mass; MTE, maternal transfer efficiency; RMML, relative maternal mass loss; TMML, total maternal mass loss.

In all models, maternal identity (γ_i_ = *N*(*0*, *δ* ^*2*^)) and study year (Y_*k*_ = *N*(*0*, *δ* ^*2*^)) were included as random effects to account for lack of independence among repeated measures of the same individual and environmental variation, respectively, that may have influenced allocation. Estimates ± standard errors (SE) are given for fixed effects.

## 3 Results

**FIGURE 2:**
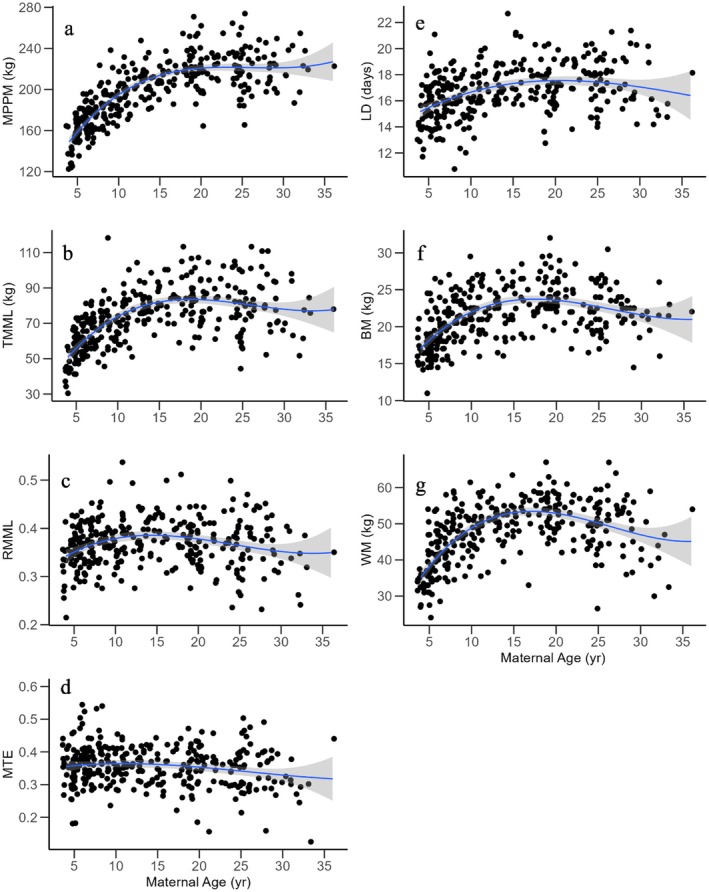
(a) Maternal postpartum body mass (MPPM), (b) total maternal mass loss (TMML), (c) relative maternal mass loss (RMML), (d) mass transfer efficiency (MTE), (e) lactation duration (LD), (f) pup birth mass (BM) and (g) pup weaning mass (WM) as a function of maternal age in grey seals at Sable Island. Blue line is the mean smoother with 95% confidence interval.

Descriptive statistics of the body mass, change in mass and lactation duration variables measured for 222 adult female grey seals and their 324 male and female offspring near birth and at weaning are given in Table 2. Female age influenced most response variables over a wide range of ages (Figure 2). MPPM increased with maternal age until about age 20, after which there was increased variation but little evidence of a trend (Figure 2a). TMML followed a similar pattern with maternal age, but there was some evidence for a decline in the oldest females (Figure 2b). RMML and MTE varied considerably with maternal age but exhibited evidence of a slight decline in females older than about 15 years (Figure 2c,d). Lactation duration was quite variable with age. Young females and those greater than about age 25 had shorter lactation lengths than prime‐age females (~15–20 years, Bowen et al. 2006; Figure 1e). Pup birth mass and weaning mass also increased with maternal age to about age 15, but unlike MPPM, declined in females aged about 25 years and older (Figure 2e,f). The top five models for each response variable plus the means model are given in Data S1 (Tables A1, A2, A3, A4, A5, A6, A7 in Appendix 1).

**TABLE 2:** Summary statistics of 222 adult female grey seals and their male and female pups measured near birth^a^ and at weaning on Sable Island, Nova Scotia.

Variable	Male pup (*n* = 145)		Female pup (*n* = 179)	
	Mean	SE	Mean	SE
MPPM (kg)	194.0	2.50	200.5	2.27
TMML (kg)	73.0	1.46	73.1	1.07
RMML	0.37	0.004	0.36	0.003
Mass transfer efficiency	0.36	0.004	0.35	0.004
Lactation duration (d)	16.7	0.16	16.8	0.14
Pup birth mass^a^ (kg)	21.8	0.30	21.3	0.25
Pup weaning mass (kg)	47.9	0.76	46.9	0.57
Maternal age (yr)	14.0	0.72	14.8	0.58

Abbreviations: MPPM, maternal postpartum mass, extrapolated to birth and used in analyses; RMML, relative maternal mass loss; TMML, total maternal mass loss.

^a^ Pup mass at first capture (average of 3d), used as proxy for pup birth mass.

### 3.1 Maternal Postpartum Mass (MPPM)

**FIGURE 3:**
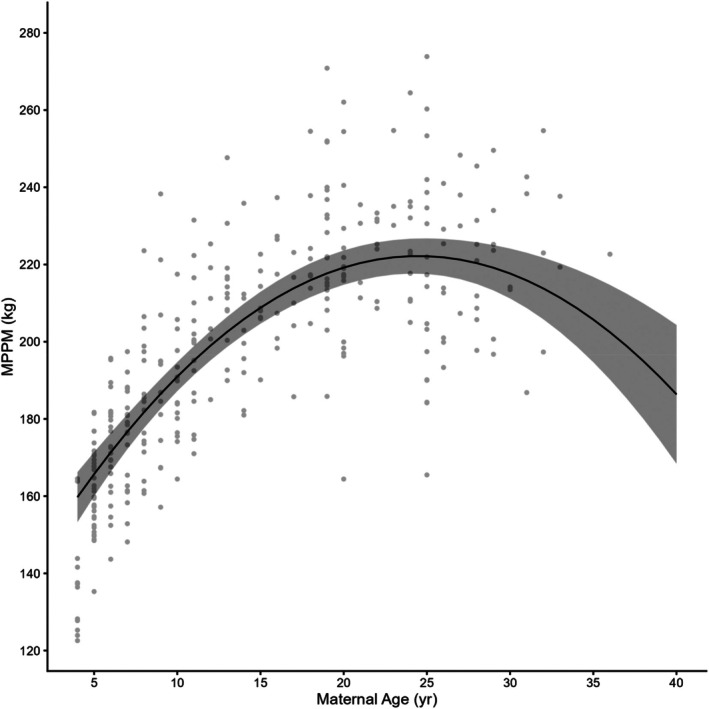
Maternal postpartum mass (MPPM) as a function of maternal age for 222 adult grey seals. Model fit is a solid black line, and the shaded area is the 95% CI.

MPPM ranged from 123 to 275 kg, with a mean of 197.6 ± 1.67 kg overall (Table 2, broken down by offspring sex). The preferred model to explain variation in MPPM, with an Akaike weight of 0.70, included maternal age as a quadratic, and parity as fixed effects (Table 3). Coefficients of the retained covariates for all preferred models of maternal response variables fit to the untransformed covariates are given in Table 4. The fixed effects explained 66.8% of the variance (*R* ^2^(*m*) = 0.668, *R* ^*2*^(*c*) = 0.918). The model fit showed that MPPM increased with female age over the first 20 years of life, but there was evidence of a decline in older females (Figure 3). MPPM also increased with parity, with first and second parity females weighing an average of −11.6 kg and −5.1 kg less than parity 3+ females (mean = 208.9), respectively. Maternal identity and study year accounted for 69.0% and 6.0% of the variance unexplained by the fixed effects, respectively, indicating a strong individual female effect but that study year had little impact on MPPM.

**TABLE 3:** Summary statistics for preferred models explaining variation in each of the eight response variables of maternal allocation in grey seals.

Model	Predictors	K	LL	AIC_c_	*w*_*i*_
MPPM (kg)	~maternal age + maternal age^2^ + parity	8	−241.77	499.99	0.70
TMML (kg)	~MPPM + pup sex + pup sex*MPPM	9	−268.45	555.47	0.81
RMML	~maternal age + pup sex + pup birth mass	7	540.57	−1064.48	0.30
MTE	~maternal age + parity + pup sex	8	451.79	−887.13	0.18
Lactation duration (d)	~maternal age + maternal age^2^ + MPPM + pup sex + pup birth mass + pup growth rate	10	−354,96	730,63	0.39
Pup birth mass (kg)	~maternal age + maternal age^2^ + parity + MPPM + pup sex + pup age	11	−378.14	779.12	0.60
Pup weaning mass (kg)	~maternal age + maternal age^2^ + TMML + parity + lactation length + pup sex + pup mass	11	−260.14	543.12	0.46

Abbreviations: MPPM, maternal postpartum mass; MTE, maternal transfer efficiency; RMML, relative maternal mass loss; TMML, total maternal mass loss.

**TABLE 4:** Regression coefficient estimates (marginal mean and standard error) for the factors influencing maternal response variables (*n* = 324) from the preferred model fit to the unscaled variables. Coefficients in boldface are those for which the 95% confidence interval does not include zero.

Parameter	MPPM (kg)	TMML (kg)	RMML	Mass transfer efficiency	Lactation duration (d)
Intercept	**135.37 (6.0)**	**−23.06 (5.37)**	**0.30 (0.02)**	**0.39 (0.01)**	**12.45 (0.74)**
Maternal age	**7.29 (0.69)**	NA	**0.01 (0.00)**	**−0.002 (0.001)**	**0.21 (0.07)**
Maternal Age	**−0.15 (0.02)**	NA	0.00 (0.00)	NA	**−0.01 (0.00)**
Parity 1	**−11.55 (3.68)**	NA	NA	**−0.03 (0.01)**	NA
Parity 2	−5.13 (3.43)	NA	NA	**0.001 (0.01) 0.03**	NA
MPPM	NA	**0.49 (0.03)**	NA	NA	**0.05 (0.01)**
Pup sex (female)	NA	**19.63 (6.72)**	−0.01 (0.005)	−0.01 (0.01)	**−0.36 (0.16)**
Pup birth mass	NA	NA	0.002 (0.001)	NA	**−0.22 (0.03)**
Pup growth rate	NA	NA	NA	NA	**−0.71 (0.22)**
Lactation duration	NA	NA	NA	NA	NA
MPPM*Pup sex	NA	**−0.11 (0.03)**	NA	NA	NA
σ_Mother_	0.69	0.35	0.27	0.20	0.38
σ_Year_	0.06	0.04	0.05	0.05	0.04
σ	0.25	0.61	0.68	0.75	0.58

Abbreviations: MPPM, maternal postpartum mass; MTE, maternal transfer efficiency; RMML, relative maternal mass loss; TMML, total maternal mass loss.

### 3.2 Total Maternal Mass Loss (TMML)

**FIGURE 4:**
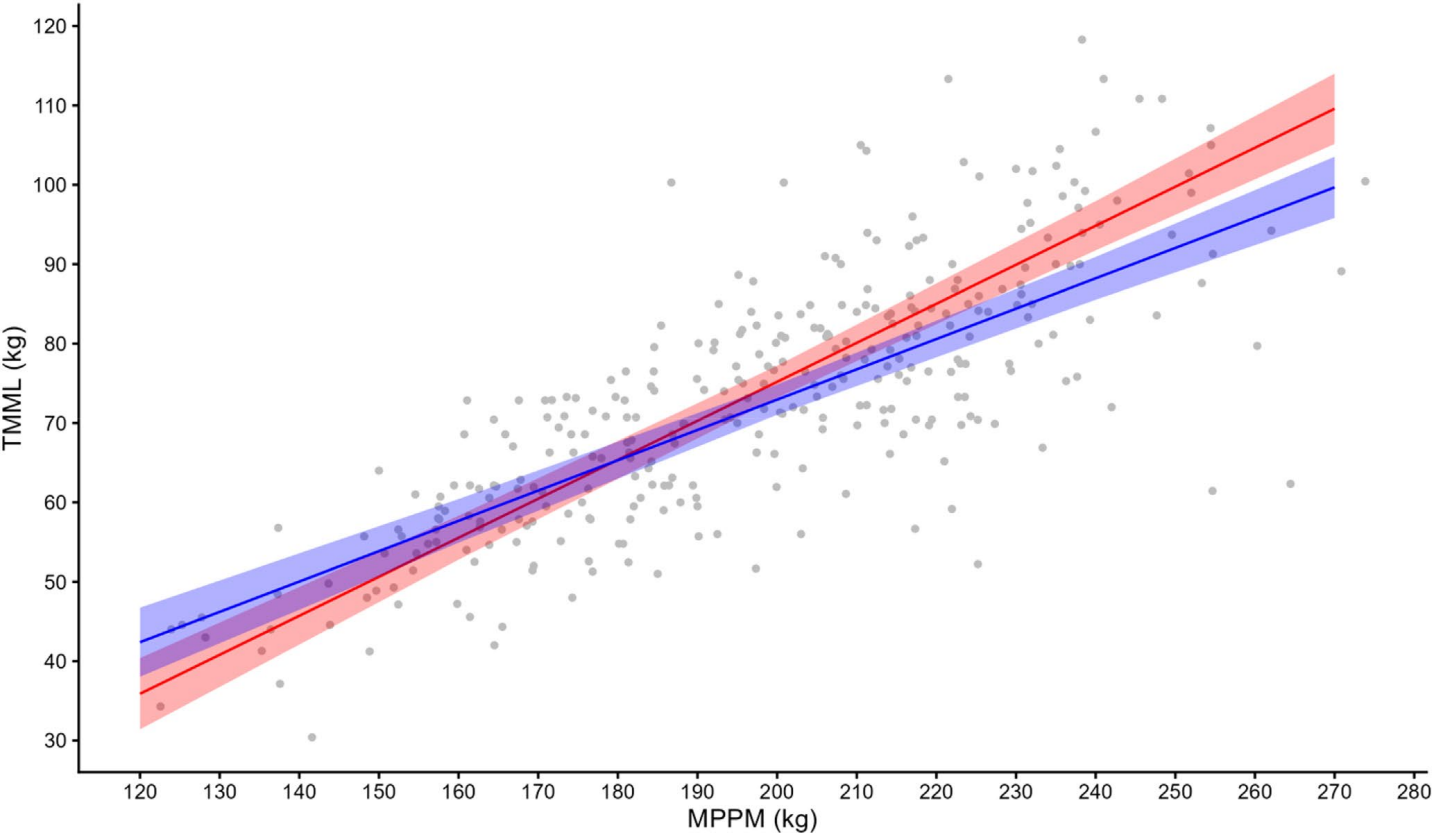
Total maternal mass loss (TMML) of 222 females plotted against maternal postpartum mass (MPPM). Predicted relationships for male (red) and female (blue) pups with 95% CI.

TMML during lactation varied four‐fold from 30 to 118 kg, with a mean of 73.1 ± 0.88 kg (Table 2, broken down by sex). The preferred model, with an Akaike weight of 0.81, included MPPM, pup sex and an interaction between MPPM and pup sex (Tables 3 and 4). The fixed effects in this model explain 64.6% of the variance (*R* ^*2*^(*m*) = 0.646, *R* ^*2*^(*c*) = 0.792). Maternal identity accounted for 35% of the variance, whereas year accounted for only 6.0%. TMML increased linearly with MPPM (Figure 4). However, there was an interaction between MPPM and pup sex showing that lighter females with female pups lost more body mass than those with male pups, but in females weighing more than about 180 kg (i.e., near the population mean), the situation was reversed such that females with male pups lost increasingly more body mass than those with female pups (Figure 4).

### 3.3 Relative Maternal Mass Loss (RMML)

RMML ranged from 0.21 to 0.54, with a mean of 0.37 ± 0.003 (Table 2, broken down by sex). The preferred model, with an Akaike weight of only 0.30, included maternal age, pup birth mass and pup sex (Tables 3 and 4). Although pup sex was retained in the model, suggesting that females that gave birth to male pups invested relatively more of their body mass than those with female pups, the biological significance of this difference (~1%) is doubtful. Similarly, the confidence limits around pup birth mass included zero indicating that the effect on RMML was negligible. Two other models also suggested mixed weak support for the influence of birth mass and pup sex on RMML (Figure 1). The fixed effects in the best model explained only 8.6% of the variance (*R* ^*2*^(*m*) = 0.086, *R* ^*2*^(*c*) = 0.375). Maternal identity and year accounted for 27% and 5.0%, respectively, of the variance unexplained by the fixed effects. RMML varied greatly but there was little evidence of a trend with maternal age (Figure 2c).

### 3.4 Mass Transfer Efficiency (MTE)

MTE varied greatly from 0.13 to 0.74, with a mean of 0.36 ± 0.003 (Table 2, broken down by sex). None of the covariates included in the models reliably predicted MTE. The best supported model (Akaike weight 0.18) retained only maternal age and parity (Tables 3 and 4). However, the fixed effects in the best supported model explained only 5.0% of the variance (*R* ^*2*^(*m*) = 0.051, *R* ^*2*^(*c*) = 0.285). There was also weak support for the next three models, which suggested some influence of pup sex and MPPM (Appendix 1). Parity and maternal age had similar sized effects on MTE but with opposite signs. Body mass transfer decreased slightly with increasing maternal age (Figure 2d), but increased slightly in females of greater reproductive experience. Maternal identity accounted for 20.0% of variance unexplained by the fixed effects in the model, whereas year accounted for just 5.0%.

### 3.5 Lactation Duration

**FIGURE 5:**
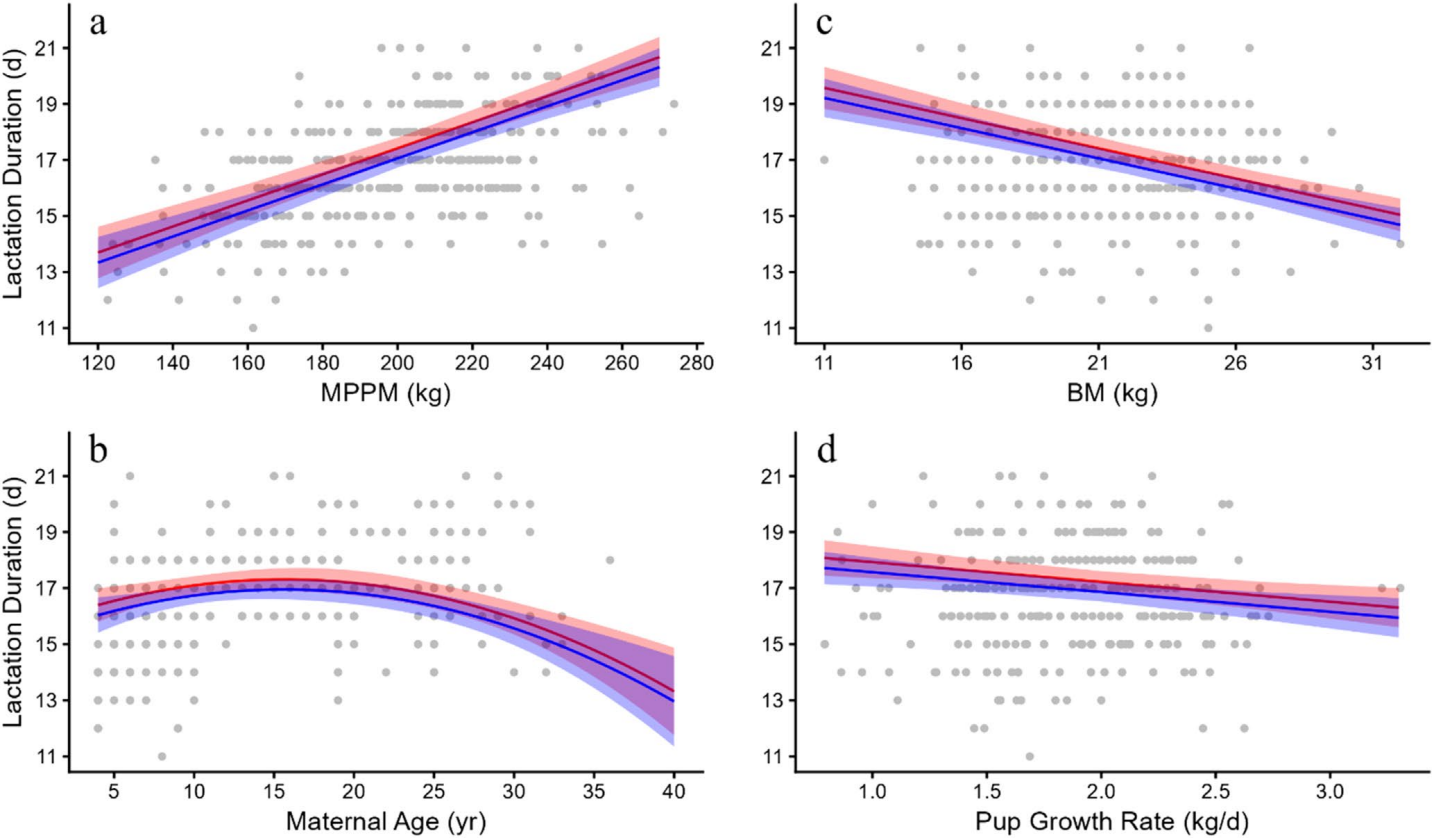
Lactation duration of females plotted against (a) maternal postpartum mass (MPPM), (b) maternal age, (c) pup birth mass (BM) and (d) pup growth rate. Predicted relationships for male (red) and female (blue) pups with 95% CI (*n* = 323). Note that the *y*‐axis of these plots begins with different values.

Lactation duration ranged from 11 to 21 days with a mean of 16.7 ± 0.10 days (Table 2, broken down by sex of offspring). Two models had similar support with Akaike weights of 0.35 and 0.32, respectively (Table 3, Appendix 1). The second supported model included the age at which the pup was weighed as a fixed factor. However, as pup age and birth mass are positively correlated, this model is not biologically relevant. The best supported model for lactation duration included maternal age as a quadratic, MPPM, pup birth mass, pup mass gain and pup sex (Table 4). The fixed effects in the model explain 42.2% of the variance (*R* ^*2*^(*m*) = 0.422, *R* ^*2*^(*c*) = 0.663). Maternal identity accounted for 38% of the variance, whereas year accounted for only 4.0%. MPPM and pup birth mass had the strongest influence on lactation duration, but their effect was opposite (Figure 5a,c). Heavier females lactated longer, a difference of about 7 days over the range of MPPM in our sample. Those with heavier newborns lactated for about 4days less than females with the lightest pups. Maternal age also influenced lactation duration, increasing slightly between ages 5 and 10 years and then declining by some 4 days in the oldest females (Figure 5b). Females that gave birth to female pups lactated 0.4 days less than females who gave birth to male pups (Table 4). Finally, females whose pups gained mass more quickly also lactated for fewer days (Figure 5d).

### 3.6 Pup Birth Mass

**FIGURE 6:**
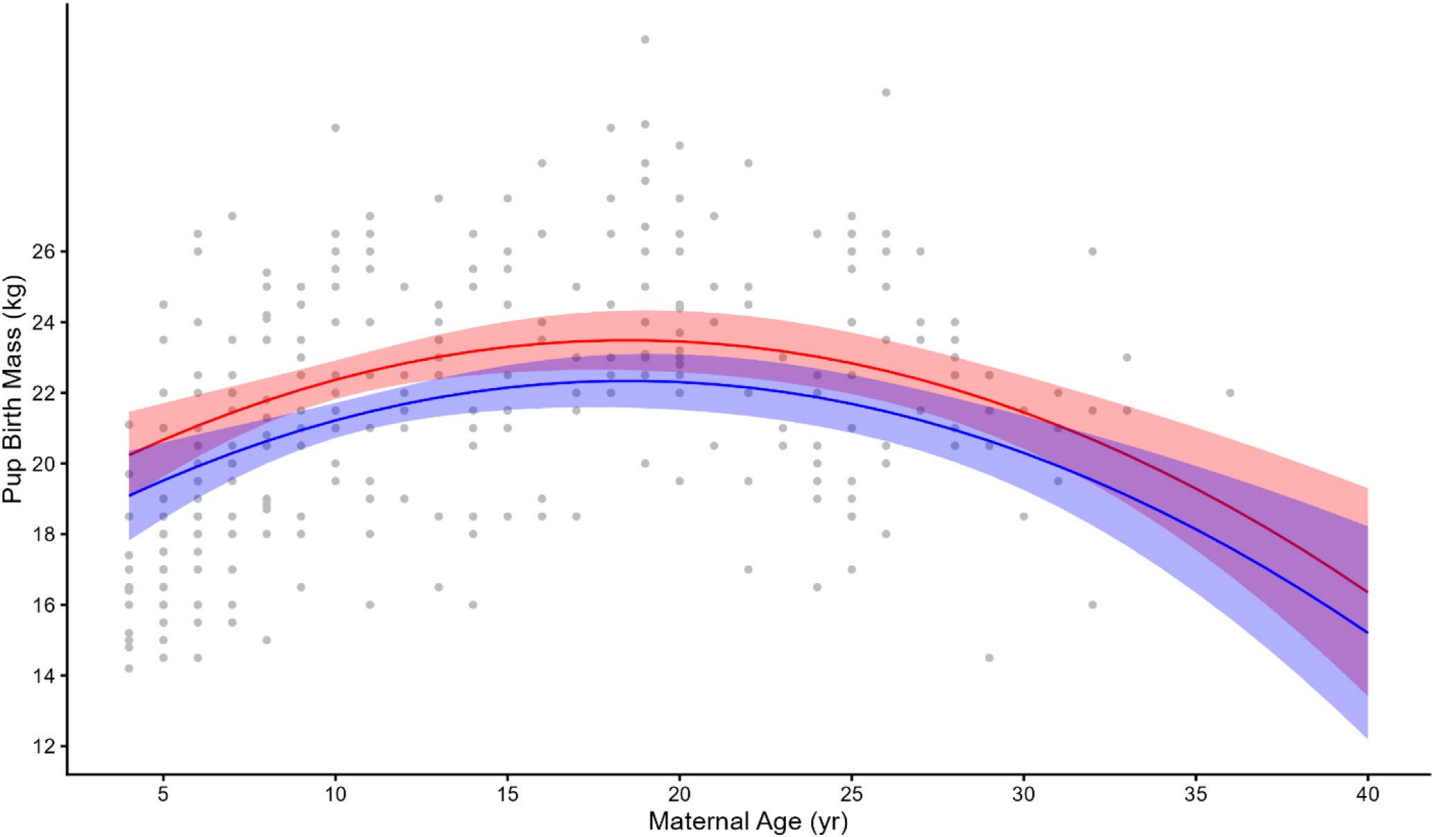
Predicted pup birth mass (Pup BM) in relation to maternal age and pup sex (red = male, blue = female) for 323 pups of 222 females. Shaded areas are the 95% CI.

Birth mass of the 324 pups varied three‐fold from 12 to 32 kg with a mean of 21.5 ± 0.20 kg (Table 2, broken down by sex). The preferred model, with an Akaike weight of 0.60, included all the covariates (Table 3). The fixed effects in the model explained 37.8% of the variance (*R* ^*2*^(*m*) = 0.37.8, *R* ^*2*^(*c*) = 42.9). Maternal identity accounted for 8% of the. Year accounted for none of the variance (Table 5). Maternal age, parity and pup sex had the strongest effects on pup birth mass. Pup age at the time of measurement was also significant but is of little relevance beyond its correlation with pup birth mass (Table 5). The relationship between pup birth mass and maternal age was dome shaped with both younger and older females giving birth to lighter pups, such that females aged 30 years and older gave birth to pups weighing the same as primiparous females (Figure 6). Female pups weighed an average of 1.2 ± 0.32 kg less than male pups at birth. Females of both parity 1 and parity 2 produced lighter pups than parity 3+ females (−2.1 ± 0.65 kg and −1.15 ± 0.66 kg lighter, respectively). However, the difference in pup birth mass between parity 2 and parity 3+ females was not significant.

**TABLE 5:** Regression coefficient estimates (marginal mean and standard error) for the factors influencing pup response variables (*n* = 324) from the preferred model fit to the unscaled variables. Coefficients in boldface are those for which the 95% confidence interval does not include zero.

Predictors	Pup body mass (kg)	
	Birth	Weaning
Intercept	**11.70 (1.94)**	**−6.79 (2.13)**
Maternal age	**0.57 (0.14)**	0.32 (0.20)
Maternal age^2^	**−0.02 (0.00)**	**−0.01 (0.01)**
Parity 1	**−2.1 (0.65)**	**−2.45 (0.97)**
Parity 2	−1.15 (0.66)	**−**0.42 (0.94)
MPPM	0.02 (0.01)	NA
Pup birth mass	NA	**0.89 (0.08)**
Pup age	**1.15 (0.40)**	NA
Pup sex	**−1.15 (0.32)**	−0.76 (0.45)
Lactation length	NA	NA
TMML	NA	**0.28 (0.02)**
σ_Mother_	0.08	0.21
σ_Year_	0.00	0.04
σ	0.92	0.75

Abbreviations: MPPM, maternal postpartum mass; TMML, total maternal mass loss.

### 3.7 Pup Weaning Mass

**FIGURE 7:**
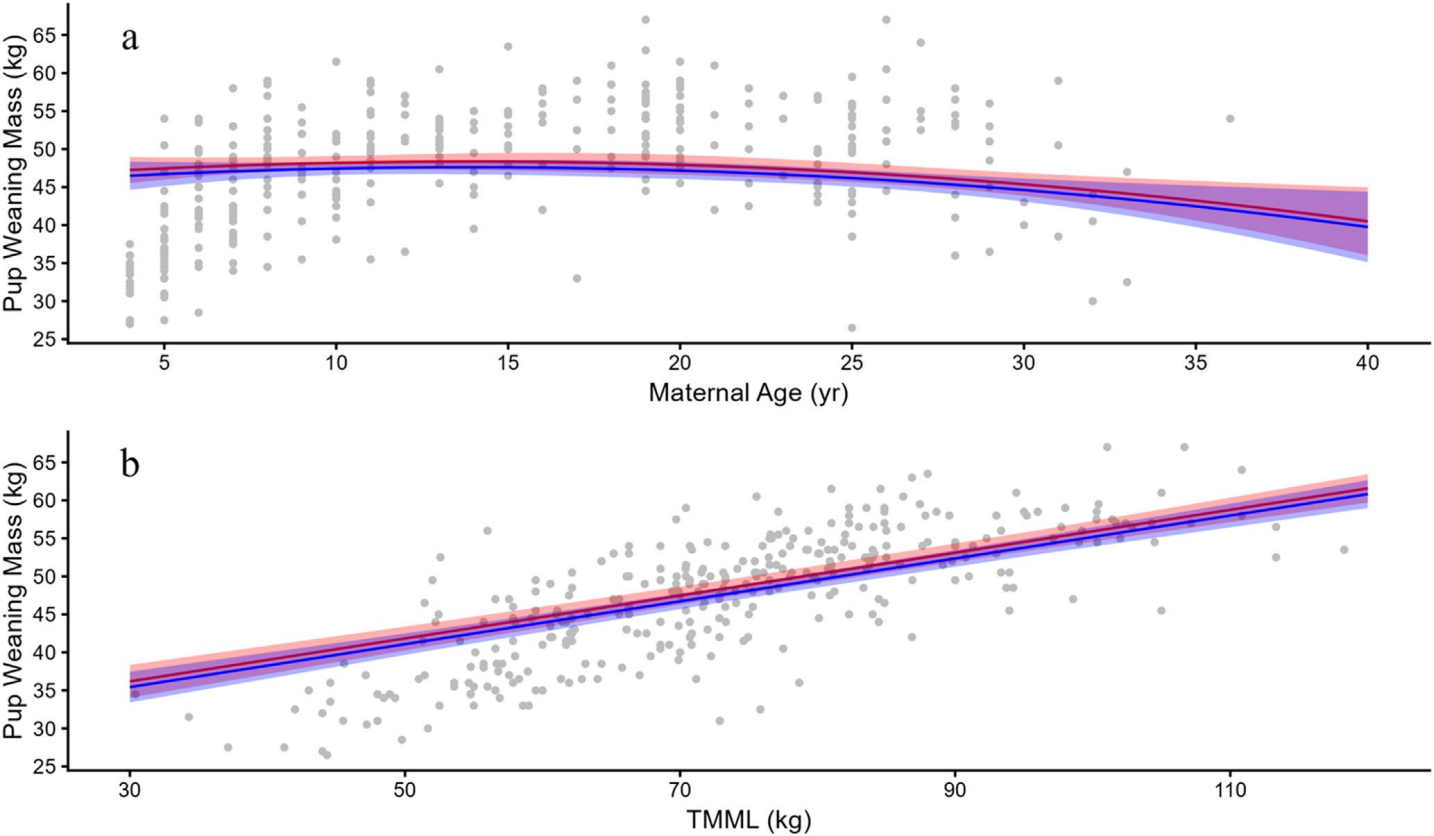
Pup weaning mass (Pup WM) in relation to (a) maternal age and (b) total maternal mass loss (TMML). Predicted relationships for male (red) and female (blue) pups with 95% CI (*n* = 323).

Weaning mass of the 323 pups ranged from 26.5–67.0 kg, with a mean of 47.4 ± 0.46 kg (Table 2, broken down by sex). Two models, one including TMML and another using MPPM, explained variation in pup weaning mass similarly well. However, as MPPM and TMML are highly correlated (*r* = 0.78) and the model with TMML fit somewhat better, we report only those results. The best supported model (Akaike weight of 0.46) included maternal age as a quadratic, TMML, parity, pup sex and pup birth mass as fixed effects (Table 5). Two other models received some support with Akaike weights of 0.25 and 0.15, differed from the best model by dropping the quadratic maternal age effect and the influence of pup sex (Appendix 1). The fixed effects in the best model explained 76.7% of the variance (*R* ^*2*^(*m*) = 0.767, *R* ^*2*^(*c*) = 0.825). Maternal identity and year as random effects accounted for 21.0% and 4.0%, respectively, of the unexplained variance (Table 5). All covariates in the preferred model (i.e., maternal age, TMML, parity, pup birth mass and pup sex) had similar influence on weaning mass (Figure 7a,b). Pup weaning mass increased linearly with pup birth mass and TMML with a 2‐fold increase over the measured range. Pup weaning mass increased with increasing parity and male pups on average were 0.76 kg heavier than female pups.

### 3.8 Influence of Previous Allocation on Current Allocation

**FIGURE 8:**
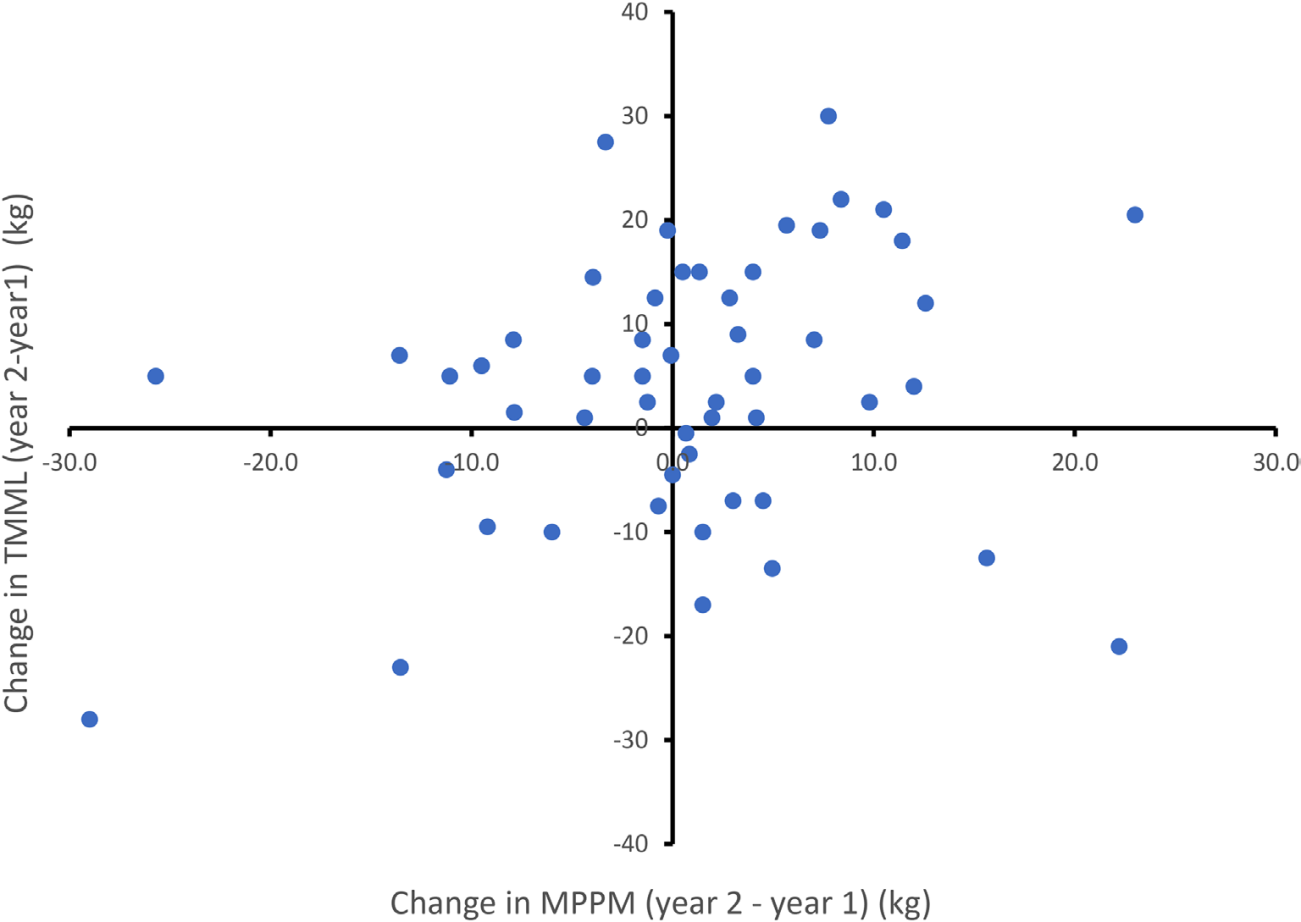
Change in total maternal mass loss (TMML) of 33 grey seal females in consecutive breeding seasons (53 paired observations) as a function of the change in maternal postpartum mass (MPPM) from year 2—year 1.

**FIGURE 9:**
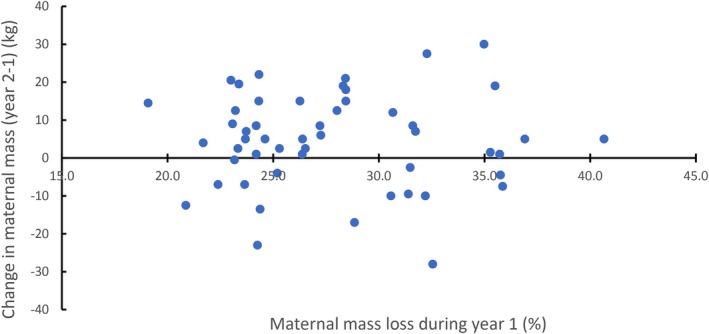
Change in maternal postpartum mass (MPPM, year 2—year 1) in relation to relative maternal mass loss (RMML, %) in year 1 of 33 grey seal females with offspring in successive years (53 paired observations).

Thirty‐three females were studied in consecutive years providing 53 observations to examine the influence of the previous year's (year 1) energy allocation to offspring on the following year's (year 2) allocation. These females covered the reproductive age range in the population (5–32 years) and all except four females were multiparous. We calculated the difference in MPPM of the females in year 2 minus year 1 and the corresponding difference in the TMML. We found a weak positive correlation (*r* = 0.28), such that females that were heavier in year 2 than in year 1 tended also increase their maternal expenditure of body mass (Figure 8). We found no relationship between the change in MPPM in year 2—year 1 and the RMML in year 1 (Figure 8). Most females (i.e., 37 of 53 or 70%) recovered or increased body mass by an average of 6.4% ± 5.3% in the second year indicating that they were able to fully recover the loss of body mass experienced during lactation in year 1 (Figure 9). This remained the case for non‐growing females 15 years and older with 13 of 19 (68%) having recovered MPPM in year 2. In these females, their allocation of body mass energy during year 1 had no discernible influence on the magnitude of allocation in year 2 (Figure 9).

## 4 Discussion

As capital breeders, grey seal females store large quantities of energy prior to parturition, mainly in the form of lipids in blubber, which are mobilised while fasting during lactation (Iverson et al. 1993). Thus, all nutrients required during lactation by both mother and offspring are brought ashore prior to parturition. Our findings indicate that during the 17‐day lactation period females expended about 37% (73 kg) of their MPPM to support their own metabolic requirements and milk production. Maternal age and MPPM had significant effects on multiple aspects of maternal energy allocation, including TMML, lactation duration, pup birth mass and pup weaning mass. Heavier females lost more body mass over lactation than lighter females. Maternal allocation increased throughout early life, plateaued in slow‐growing prime‐age females, and then declined in older females. Parity also affected maternal allocation, but the effect was limited to young females, with influences on pup birth mass, proportional pup mass gain and weaning mass. Females that lactated longer produced pups with greater RMML and weaning mass. Despite the substantial loss of body mass during lactation, 33 females measured in 2 consecutive years recovered fully from this energy expenditure and we found no evidence that females breeding in the following year reduced allocation compared with that in the previous year.

Studies on both aquatic (Weddell seals, *Leptonychotes wedellii* [e.g., Wheatley et al. 2006]; and southern and northern elephant seals, *Mirounga leonine* and *M. angustirostris* [e.g., Fedak et al. 1996; Crocker et al. 2001]) and terrestrial (e.g., reindeer, *Rangifer tarandu* [Wedalji et al. 2020]; polar bears, *Ursus maritimus* [Derocher and Stirling 1998]; mountain goats, *Oreanmnos americanus* [e.g., Arnbom et al. 1997: Hamel et al. 2010]; and bison, *Bison bison* [Hamel et al. 2012]) species have found that many of the same traits influence maternal allocation. Findings from these diverse taxa underscore the importance of these traits in the evolution of capital breeding strategies.

Maternal identity, as a random effect, accounted for 69% of the residual variance in offspring weaning mass and 8%–38% for other response variables, suggesting that characteristics of maternal allocation are repeatable to a varying extent within individual females. Repeatability measures the proportion of phenotypic variation in a trait that is attributable to either genetic or permanent environmental effects among individuals and can provide an upper bound to heritability (Falconer 1996). High levels of within‐female repeatability in grey seals are also evident in milk composition, daily milk output, lactation duration, personality (i.e., boldness), birth dates and breeding frequency (Pomeroy et al. 1999; Lang et al. 2009; Bubac et al. 2018; Bowen et al. 2020; Badger et al. 2023). This has also been observed in other long‐lived species, such as Weddell seals (Mannus 2011; Paterson et al. 2016) and bison (Hamel et al. 2012). Thus, components of maternal allocation may be heritable traits of individual females and therefore subject to selection. By contrast, year as a random effect explained little (0%–6%) of the residual variation of response variables suggesting that environmental variation during our study had little influence on maternal allocation. However, the lack of a year effect could also reflect the fact that the population was increasing and therefore was likely experiencing a favourable environment with little variation during our study (Rossi et al. 2021; Hammill et al. 2023). In any case, our conclusions about the influence of random effects are tentative as only 27.5% of the females had measurements in more than 1 year. Therefore, we may have underestimated among‐individual variability in components of allocation given the strong influence of environmental variation (i.e., year effects) on birth dates in this population (Bowen et al. 2020). Furthermore, environmental variation has been shown to influence maternal allocation in other mammals (McMahon et al. 2017; MacKay et al. 2018).

There are several other limitations to this study, despite the relatively large number of females and their offspring sampled. First, we sampled a large fraction of young females (71%) in the first 5 years of the study, as these were the only individually marked females in the population. This could explain the smaller sex difference in pup weaning masses in this study compared with that found in previous studies of this population (Bowen et al. 2006) and this bias could have impacted the effect of other covariates on response variables. Second, the effect of environmental variation on maternal energy allocation could not be examined in detail because there were few samples in many years, and there was a strong correlation between year and maternal age that confounded any year effect.

Our findings indicate that maternal age was an important predictor of MPPM and most components of maternal energy allocation in grey seals, but the effect of age changed over the life of females (Figure 1). Allocation increased with age in young females but decreased with age in older females, providing evidence of senescent decline. This confirms conclusions from an earlier study on the same population (Bowen et al. 2006). Improvement in reproductive performance with maternal age appears to be widespread in mammals, including pinnipeds (Reiter et al. 1981, Ellis et al. 2000; Crocker et al. 2001; Bowen et al. 2006; Mannus 2011; Paterson et al. 2016), whales (Olesiuk et al. 1990), polar bears (Derocher and Stirling 1998), primates and lions (Packer et al. 1998), ungulates (Clutton‐Brock et al. 1982; Bercovitch et al. 1998; Ericsson and Wallin 2001; Wedalji et al. 2020) and birds (Robertson and Rendall 2001; Weimerskirch 1992).

Senescent decline in components of energy allocation has also been reported among diverse vertebrate species (Nussey et al. 2013), including both terrestrial and aquatic mammals (Bowen et al. 2006; Hamel et al. 2012; Festa‐Bianchet et al. 2019; Condit et al. 2014; Paterson et al. 2016; Lee et al. 2016; Naciri et al. 2022). Senescent decline in prenatal investment observed in grey seals and Weddell seals (Mannus 2011) may be the result of increased maternal maintenance costs (Testa et al. 1989; Pomeroy et al. 1999), a decline in uterine and placental function (Wilsher and Allen 2003), reduced foraging efficiency, reduction of nutrients transferred to the foetus (Bowen et al. 2006), or some combination of these mechanisms. By contrast, the decline in offspring size at weaning (i.e., postnatal allocation) with maternal age presumably is associated with a decline in the physiological capacity associated with milk production (Lang et al. 2009). Although clearly present in grey seals, there is little evidence for senescent decline in weaning mass in Weddell seals (Mannus 2011, Macdonald et al. 2020).

Patterns of maternal energy allocation have been shown to differ during the prenatal and postnatal period (Lock et al. 2007; Weladji et al. 2010) because these two periods reflect the different physiological processes of gestation and lactation (Oftedal 1985). However, this appears not to be the case in grey seals, as both prenatal (i.e., pup birth mass) and postnatal (pup weaning mass) allocation varied with age as a quadratic, increasing with MPPM and parity, and differed by pup sex. In Weddell seals, younger females increased allocation with age in both periods, but older females exhibited a senescent decline in offspring birth mass, as in grey seals, followed by an increase in energy allocation during the postnatal period (Paterson et al. 2016) that was not observed in grey seals. Thus, in both species the pattern of energy allocation during the prenatal period supports the constraint hypothesis whereby females acquire or improve skills or physiological function with age; (Forslund and Part 1995). During the postnatal period, Weddell seal data support the terminal allocation hypothesis, whereby females are predicted to increase their investment in current reproduction to maximise their remaining lifetime reproductive success given a decreased expectation of survival, (Williams 1966). On the other hand, our grey seal data are consistent with the disposable soma hypothesis (Bowen et al. 2006). This hypothesis predicts that accumulation of random molecular and cellular damage limits maintenance and repair resulting in reduced function (Kirkwood and Austad 2000).

A previous study of this population found no evidence of a decline in MPPM in older females (Bowen et al. 2006) suggesting that, in contrast with some birds and other mammals (Weimerskirch 1992; Broussard et al. 2005; Proffitt et al. 2007; Paterson et al. 2016), there was no evidence of body mass senescence in grey seals. However, with more observations of females aged 25+ than available in Bowen et al. (2006), the quadratic age term was retained in the model suggesting evidence of a decline in MPPM of older females (Figure 2). Thus, like Weddell seals (Proffitt et al. 2007), grey seals do appear to experience senescent decline in MPPM.

Maternal body mass is likely a good measure of body condition and body energy stores (Fedak et al. 1996, Boyd 2000, Hanson et al. 2019, Crocker et al. 2001). Given the strong relationship between MPPM and body protein and lipid content in grey seals (Mellish et al. 1999), it is reasonable to assume that heavier mothers have more available resources to contribute to neonates (Iverson et al. 1993, Mellish et al. 1999, Hanson et al. 2019). Thus, grey seal females of the same age with lower postpartum energy stores wean lighter pups (Iverson et al. 1993, Mellish et al. 1999, Pomeroy et al. 1999, this study).

As RMML accounts for differences in MPPM, it should be a more informative indication of energy allocation to offspring (Hamel et al. 2012). We found that grey seal females lose on average 37% of MPPM, comparable to previous estimates for this population (39%; Iverson et al. 1993) and for grey seals on North Rona, Scotland (38%–39%; Fedak and Anderson 1982, Anderson and Fedak 1987), northern elephant seals (42%; Costa et al. 1986) and southern elephant seals (35%–37%; McCann et al. 1989; Arnbom et al. 1997). In each of these species, a third or more of body mass is lost during lactation, underscoring the large energy expenditure that these species allocate to offspring. Heavier mothers ought to be capable of allocating a greater proportion of mass to offspring (Gittleman and Thompson 1988), however, there is substantial variation among capital breeders with respect to relative allocation. In Weddell seals (Wheatley et al. 2006; Macdonald et al. 2020) and mountain goats (Cote and Festa‐Bianchet 2001), heavier females exhibit greater relative allocation than lighter ones. By contrast, studies on bison (Hamel et al. 2012) and harbour seals ( *Phoca vitulina* , Bowen et al. 2001) show that lighter mothers invest more relative mass than heavier mothers. In this study, we found that relative energy allocation increased with MPPM but that the effect was small and of doubtful biological significance, confirming previous findings on grey seals in the northeast Atlantic (Fedak et al. 1996).

Pup mass gain depends on how milk energy intake is partitioned between pup metabolism, energy storage and growth. As offspring mass at weaning is positively related to survival in many species, including pinnipeds (Hall et al. 2001; Proffitt et al. 2008; Bowen et al. 2015), a high efficiency of mass transfer from mother to offspring should be favoured. In grey seals 37%–40% of maternal mass loss is gained by pups, that is, MTE (Mellish et al. 1999, Pomeroy et al. 1999, this study). However, a higher proportion is gained in other phocids studied—Weddell seals (49.6%, Macdonald et al. 2020), hooded seals, *Cystophora cristata* , (76%, Bowen et al. 1987), southern elephant seals (46%–54%, Arnbom et al. 1997, Carlini et al. 1997) and northern elephant seals (53%–58%, Costa et al. 1986, Crocker et al. 2001). These data suggest that grey seals have less MTE than other capital breeding pinnipeds, although Baker et al. (1995) reported an MTE of 49% in ice‐breeding grey seals. It is not clear why grey seals are less efficient.

Sexual dimorphism at weaning has been documented for many mammals (grey seals; Hall et al. 2001, red deer; Froy et al. 2016, big horn sheep; Hogg et al. 1992, harbour seals; Bowen et al. 1994, California sea lions; Ono and Boness 1996). Male pups have been hypothesised to be more energetically expensive than female pups (Festa‐Bianchet et al. 2019). Our results indicate that male grey seal pups receive greater prenatal and postnatal allocation than female pups; however, the differences were small. Maternal allocation to male pups was greater than to female pups for all response variables measured except for TMML (Table 5). Male pups were 0.75 kg heavier than female pups at weaning; in comparison, previous studies on grey seals found male pups to weigh ~2 kg more than female pups at weaning. As noted earlier, this may be due to the large proportion of young females included in this study.

We found that parity was a useful predictor of MPPM, pup birth and weaning mass, and MTE, although in this latter case the effect was small (Table 3). Primiparous females were lighter and allocated less energy than second‐time breeders or multiparous females that had the highest level of allocation, as reported in Bowen et al. (2006). Studies on capital breeders and other mammals have found similar results (Lang et al. 2009; Hamel et al. 2012; Festa‐Bianchet et al. 2019). One proposed mechanism is that primiparous females have less developed secretory cells in the mammary gland, and in general the cells may have less secretory activity throughout lactation than multiparous females (Lang et al. 2012). Primiparous grey seal females do have significantly lower daily milk output than multiparous females (Lang, Iverson, and Bowen 2011). Growth of mammary gland structural tissues through juvenile life up to the first reproductive event is correlated strongly with body size in grey seals (Cowie et al. 1980). Thus, females that are larger at their first reproductive event have larger mammary glands and greater milk output, which may explain some of the observed variation with parity (Lang, Iverson, and Bowen 2011). Another proposed mechanism is that females begin to reproduce before reaching full adult size, and therefore first‐time breeders usually have lower offspring growth rates and weaning mass (Lang, Boness, et al. 2011) because of the trade‐off between maternal growth and reproduction. Finally, as lactation behaviours take time to develop, multiparous females with more experience show improvement in quality and intensity of maternal care than primiparous females (Timmermans and Vossen 1996; Numan et al. 2006). However, previous research on grey seals has ruled out reduced maternal care behaviours as the cause for decreased primiparous performance (Lang, Iverson, and Bowen 2011).

Lactation in capital breeding species involves substantial loss of body mass during a period of high energy expenditure. Thus, it is possible that energy allocation during one lactation may negatively affect the level of allocation during the following lactation period if the energy and nutrients needed to successfully wean offspring have not been recovered (Creighton et al. 2009). However, we found no evidence that the 33 grey seal females sampled in consecutive lactations exhibited reduced energy allocation in the current year. On average these females fully recovered body mass and there was no correlation between previous allocation and offspring weaning mass in the subsequent year. Furthermore, Badger et al. (2023) found no evidence to support a trade‐off in reproductive performance and survival at the individual female level suggesting that individual maternal quality is a stronger driver in life history variation than individual strategies resulting from energetic trade‐offs (i.e., a cost of reproduction). A larger sample of females may provide evidence for a relationship between level of allocation and weaning mass, but for now our results differ from the negative relationship reported for grey seals in the UK (Pomeroy et al. 1999) and for several other species (Hamel et al. 2011; Festa‐Bianchet et al. 2019; Hanson et al. 2019).

## Author Contributions

**M. Sanchez:** formal analysis (lead), investigation (equal), writing – original draft (lead), writing – review and editing (equal). **W. D. Bowen:** conceptualization (lead), formal analysis (equal), funding acquisition (lead), investigation (equal), methodology (lead), writing – original draft (lead), writing – review and editing (equal). **C. E. den Heyer:** data curation (lead), formal analysis (lead), funding acquisition (supporting), investigation (equal), methodology (equal), writing – review and editing (equal). **S. J. Iverson:** funding acquisition (supporting), investigation (equal), methodology (equal), writing – review and editing (equal).

## Funding

This work was supported by the Natural Sciences and Engineering Research Council of Canada (WDB 05403‐18) and Fisheries and Oceans Canada.

## Ethics Statement

All procedures used on study animals complied with applicable animal care guidelines of the Canadian Council on Animal Care and were approved by The Department of Fisheries and Oceans Animal Care Committee and the Dalhousie University Animal Care Committee.

## Conflicts of Interest

The authors declare no conflicts of interest.

## Supporting information


**Data S1:** Supporting Information.

## Data Availability

All data are provided in the accompanying csv file (Sanchez_2025.csv).

## Appendix 1

**TABLE A1:** Top five best fitting models plus the intercept only model to explain variation in maternal postpartum mass (kg).

Model	df	LL	AIC_c_	ΔAIC_c_	*w*_*i*_
~Maternal age + Maternal age^2^ + Parity	8	−1342.13	2700.71	0	0.70
~Maternal age + Maternal age^2^ + Parity + Pup sex	9	−1342.12	2702.81	2.10	0.24
~Maternal age + Maternal age^2^	6	−1347.01	2706.30	5.58	0.04
~Maternal age + Maternal age^2^ + Pup sex	7	−1347	2708.35	7.63	0.02
~Maternal age + Parity	7	−1370.68	2755.72	55.01	0.00
Intercept only	4	−1496.97	3002.08	301.36	0.00

**TABLE A2:** Top five best fitting models plus the intercept only model to explain variation in total maternal mass loss (kg) over lactation.

Model	df	LL	AIC_c_	ΔAIC_c_	*w*_*i*_
~MPPM + Pup sex + MPPM:Pup sex	7	−1180.12	2374.59	0.00	0.81
~MPPM + Parity + Pup sex + MPPM:Pup sex	9	−1179.62	2377.83	3.23	0.16
~MPPM + Pup sex	6	−1185.23	2382.73	8.14	0.01
~MPPM	5	−1186.78	2383.75	9.16	0.01
~MPPM + Parity + Pup sex	8	−1184.59	2385.65	11.06	0.00
Intercept only	4	−1330.61	2669.35	309.14	0.00

Abbreviation: MPPM, maternal postpartum mass.

**TABLE A3:** Top five best fitting models plus the intercept only model to explain variation in relative maternal mass loss (proportion) over lactation.

Model	df	LL	AICc	ΔAICc	*w*_*i*_
~Maternal age + Maternal age2 + PupMass1 + Pup sex	8	540.57	−1064.68	0.00	*0.30*
~Maternal age + Maternal Age2 + PupMass1	7	539.23	−1064.1	0.58	*0.22*
~Maternal Age + Maternal Age2 + PupSex	7	538.77	−1063.19	1.50	*0.14*
~Maternal Age + Maternal Age2 + PupAge1 + PupMass1 + PupSex	9	540.59	−1062.6	2.08	*0.1*
~Maternal Age + Maternal Age2 + PupAge1 + PupMass1	8	539.23	−1061.01	2.67	*0.08*
Intercept only	4	527.57	−1047.01	17.71	*0.00*

Abbreviations: PupAge1, age in days at first measurement; PupMass1, first measurement of pup body mass.

**TABLE A4:** Top five best fitting models plus the intercept only model to explain variation in length of lactation (days).

Model	df	LL	AIC_c_	ΔAIC_c_	*w*_*i*_
~Pup growth rate + MPPM + Maternal Age + Maternal Age^2^ + PupMass1 + PupSex	10	−558.36	1137.42	0.00	0.35
~Pup growth rate + MPPM + Maternal Age + Maternal Age^2^ + PupAge1 + PupMass1 + PupSex	11	−557.36	1137.56	0.14	0.32
~Pup growth rate + MPPM + Maternal Age + Maternal Age^2^ + PupAge1 + PupMass1	10	−559.56	1139.83	2.40	0.10
~Pup growth rate + MPPM + Maternal Age + Maternal Age^2^ + PupMass1	9	−561.02	1140.61	3.19	0.07
~Pup growth rate + MPPM + Maternal Age + Maternal Age^2^ + Parity + PupMass1 + PupSex	12	−557.985	1140.98	3.55	0.06
~Intercept only	4	−640.60	1289.32	153.56	0.00

Abbreviations: MPPM, Maternal Postpartum Mass; PupAge1, age in days at first measurement; PupMass1, first measurement of pup body mass.

**TABLE A5:** Top five best fitting models plus the intercept only model to explain variation in birth mass of grey seal pups.

Model	df	LL	AIC_c_	ΔAIC_c_	*w*_*i*_
~MPPM + Maternal Age + Maternal Age^2^ + Parity + PupAge1 + PupSex	11	−787.00	1596.84	0.00	0.62
~Maternal Age + Maternal Age^2^ + Parity + PupAge1 + PupSex	10	−788.83	1598.36	1.54	0.29
~MPPM + Maternal Age + Maternal Age^2^ + Parity + PupSex	10	−790.94	1602.58	5.75	0.03
~MPPM + Maternal Age + Maternal Age^2^ + PupAge1 + PupSex	9	−792.16	1602.89	6.05	0.03
~Maternal Age + Maternal Age^2^ + Parity + PupSex	9	−792.94	1604.45	7.61	0.01
~Intercept only	4	−858.77	1725.67	128.82	0.00

Abbreviations: MPPM, Maternal Postpartum Mass; PupAge1, age in days at first measurement.

**TABLE A6:** Top five best fitting models plus the intercept only model to explain variation in weaning mass of grey seal pups.

Model	df	LL	AIC_c_	ΔAIC_c_	*w*_*i*_
~Maternal Age + Maternal Age^2^ + Parity + PupMass1 + PupSex + TMML	11	−898.86	1820.57	0.00	0.34
~Maternal Age + Maternal Age^2^ + Parity + PupMass1 + TMML	10	−900.26	1821.22	0.65	0.25
~Maternal Age + Parity + PupMass1 + TMML	9	−901.84	1822.25	1.68	0.15
~Maternal Age + Parity + PupMass1 + PupSex + TMML	10	−901.11	1822.92	2.35	0.11
~Maternal Age + Age^2^ + PupMass1 + PupSex + TMML	9	−902.46	1823.50	2.93	0.08
~Intercept only	4	−1115.48	2239.08	431.98	0.00

Abbreviations: PupAge1, age in days at first measurement; PupMass1, first measurement of pup body mass; TMML, Total Maternal Mass Loss.

**TABLE A7:** Top five best fitting models plus the intercept only model to explain variation in MTE.

Model	df	LL	AIC_c_	ΔAIC_c_	*w*_*i*_
~Age + Parity + PupSex	8	451.79	−887.13	0.00	0.18
~Age + Parity	7	450.72	−887.08	0.04	0.17
~MPPM + Age + Parity + PupSex	9	452.62	−886.67	0.45	0.14
~MPPM + Age + Parity	8	451.41	−886.36	0.76	0.12
~MPPM + Age + PupSex	7	450.05	−885.75	1.38	0.09
~Intercept model	4	444.65	−881.17	5.96	0.00

Abbreviation: MPPM, maternal postpartum mass.
